# The effect of stress coping styles on empathy level in students of medicine: A cross-sectional study

**DOI:** 10.1097/MD.0000000000032066

**Published:** 2022-11-25

**Authors:** Betül Kurtses Gürsoy

**Affiliations:** a Afyonkarahisar Health Sciences University, Faculty of Medicine, Department of Psychiatry, Turkey.

**Keywords:** clinical empathy, coping skills, medical education

## Abstract

We aimed to investigate the change in the ability of clinical empathy, which has a special importance in physician-patient relationship, during medical school years, and its relationship between stress coping styles. After the preliminary interview with 292 volunteer medical school students, the students were asked to answer the Hospital Anxiety Depression Scale, the Stress Coping Scale, and the student version of the Jefferson Doctor Empathy Scale. This study shows that the lowest median of the empathy level among medical school students was in the sixth year, and the decrease in empathy in the sixth year was mostly in the perspective taking component. When the relationship between empathy and coping styles with stress was examined, it was seen that self-confident approach was positively correlated with perspective taking (*R* = 0.182, *P* = .002) and standing in the patient’s shoes (*R* = 0.172, *P* = .003). It was observed that the helpless approach, which is one of the negative coping styles, was inversely correlated with standing in the patient’s shoes. As a result of the study, it was determined that the styles of coping with stress were related to the components of empathy, except for compassionate care. The self-confidence approach has an impact on the ability of standing in the patient’s shoes and perspective-taking. During medical education, focusing on the approaches that increase the student’s self-confidence against the stress will encounter throughout their professional life will undoubtedly increase the level of empathy.

## 1. Introduction

The physician’s empathy is important in recognizing the symptoms of the patients better, thus making a more accurate diagnosis, reducing the health care costs, improving the quality of care, and adapting the patients to the treatment.^[1,2]^ Besides, in a physician with developed empathy skills, it is claimed that burnout feelings will decrease with the increase in the satisfaction of the job.^[3]^ Therefore, without a doubt, one of the skills expected to be acquired in medical education is the ability to empathize. Empathy has 2 components, cognitive empathy, which is the ability to understand the feelings and experiences of other people, and emotional empathy, which is the ability to emotionally share the experiences of others.^[4]^ Empathy in physician-patient communication is called “clinical empathy.” Clinical empathy is a skill that allows doctors to understand their patients’ emotions, conditions, and perspectives and to communicate and act without adopting patients’ emotions.^[5]^

Unexpectedly, day by day, there is an increase in the studies showing that clinical empathy skill, which has special importance in physician-patient relationship, decrease during medical education.^[6–8]^ Many factors such as prejudices, patient contact, practical skills, working conditions, time pressure, individual characteristics of the student, as well as the education curriculum were held responsible for the reasons for the decrease in clinical empathy during education.^[9]^ Previously, it was thought that empathy skills could be improved with the training of communication skills, but later it has been shown by studies that communication skills courses given in medical education did not cause a significant increase in empathy skills.^[10,11]^ Although the training of communication skills given affects improving the perspective-taking behavior, it is accepted that especially compassionate care behavior, that is, the feeling of mercy is pushed into the background by the students.^[11]^ Also, it has been suggested that the effect of didactic courses on the development of empathy is limited, and the courses in which real patients are evaluated simultaneously with students are more effective in the development of empathy skills.^[12]^

Another factor contributing to the decrease in empathy level is stress. In a recent study, it was found that, during medical education, stress increased especially after the 3rd grade with the start of clinical education, but stress tended to decrease again in the following years with the adaptation process.^[13]^ Therefore, the reason for the decrease in the empathy level of students after clinical rotation can be attributed to stress and its components.^[5]^ By its nature, being a physician is a profession done under stress. Gaining a load of knowledge, applying the acquired knowledge in the clinic, any mistakes made are irreversible, and the difficulty of working conditions are just a few of the stressful aspects of medicine. However, the defense mechanism of each person against this stress is different. The style of coping with stress is a variable that shows the adaptation of the person when faced with stress. People who can control their anxiety under stress adopt approaches that are self-confident, optimistic, and seek community support.^[14]^

In the review of literature, although stress was thought to be an empathy factor, no study was found that investigated the relationship between the level of empathy and styles of coping with stress in medical students. The current study aimed to investigate the effect of styles of coping with stress on the empathy level in medical school students. The study hypothesizes that the students who adopt the methods of coping with maladaptive stress have a lower level of empathy. As a result of the study, it is expected that the level of empathy will decrease with the increase in the levels of maladaptive defense adopted towards the sixth grade.

## 2. Methods

The Afyonkarahisar Health Sciences University Faculty of Medicine Ethics Committee approved the study on 03.12.2021 with reference number #2021/548. Data collection period was determined as 15.12.2021 to 15.02.2022. After obtaining the approval of the ethics committee, an announcement was made to the 1st, 3rd, 5th, and 6th year students about the study and the volunteer students whom signed the informed consent about the study, were taken to a quiet room to fill in the scales. The volunteers included in the study were asked to answer the sociodemographic data form prepared by the researchers, the Hospital Anxiety Depression Scale, the Stress Coping Style Inventory, and the student version of the Jefferson Physician Empathy Scale. Assessment scales were given to the students in hard copy. There was no time limit for students to fill in the scales. It was determined that the participants completed the scales in an average of 1 hour. 300 medical students were included in the study, and 8 students were excluded from the study because they filled in the scales incompletely. At the end of the study, students with high depression and/or anxiety levels were taken to psychiatric interview and their treatment was planned if necessary.

### 2.1. Evaluation tools

#### 1.2.1. The sociodemographic data form

It is a form created by the researchers to obtain information about the sociodemographic characteristics of the individuals participating in the study. The form has information such as age, gender, and the year of the student.

#### 2.2.1. Hospital anxiety depression scale

It is a self-report scale with 7-item anxiety and depression subscales, developed by Zigmond and Snaith.^[15]^ As a result of the Turkish validity and reliability study conducted by Aydemir et al, the cutoff score was found 10 to 11 for the Hospital Anxiety Depression Scale/Anxiety Sub-Scale and 7 to 8 for the Hospital Anxiety Depression Scale/Depression Sub-Scale.^[16]^

#### 3.2.1. The stress coping style inventory

The scale that Şahin et al (1992) developed regarding the Folkman and Lazarus’ Ways of Coping Inventory is a self-report scale with 4 options, 30 items, and 5 subscales. Subscales include self-confident approach, optimistic approach, helpless approach, submissive approach, and searching social support approach.^[17]^

#### 4.2.1. The student version of the Jefferson Physician Empathy Scale

The student version of the Jefferson Doctor Empathy Scale was used to examine medical students’ attitudes toward the empathic physician-patient relationship, proved by Hojat et al^[18]^ that it can be used as a measurement tool in medical education. The Turkish validity and reliability of the scale were performed by Gonullu and Oztuna.^[19]^ The scale has 3 subscales called “perspective taking,” “compassionate care” and “standing in the patient’s shoes.”

### 2.2. Statistical analysis

The data were evaluated with the SPSS version 25 package program (SPSS Inc., Chicago, IL). Shapiro–Wilk test was used to determine the distribution characteristics of the variables. Continuous variables that did not show normal distribution were expressed as median and percentile ranges (Q1–Q3). The Mann–Whitney *U* test was used for comparisons of data between 2 groups that did not fit a normal distribution, the Kruskal–Wallis test was used for comparisons of 3 groups and above. The linear relationship between the scales was analyzed using the Spearman Correlation Test. A *P* value < .05 was considered statistically significant.

## 3. Results

A total of 292 students were included in the study, 182 (62.3%) were female. Ninety-nine (33.9%) students were first-year students, 51 (17.5%) were third-year students, 87 (29.8%) were fifth-year students, and 55 (18.8%) were sixth-year students.

It was observed that in the perspective-taking scale, a subscale of the empathy scale, there was a decreasing trend from the first year to the sixth year. It was determined that the fifth and sixth years got statistically significantly lower scores than the first and third years (P_6-3_ < .001, P_6-1_ < .001, P_5-3_ = .047, P_5-1_ = .001). In terms of compassionate care, it was seen that the sixth year had the lowest scores, and when compared to the other years, there was a statistically significant difference between all of them (P_6-5_ = .010, P_6-3_ < .001, P_6-1_ < .001). While the scores for standing in the patient’s shoes did not show a significant difference between years, it was observed that the lowest scores belonged to the sixth year on the total empathy scale (Table 1).

**Table 1:** Scores of JSPE-S version, styles of coping inventory and HADS.

		Year 1 median (Q_1_–Q_3_)	Year 3 median (Q_1_–Q_3_)	Year 5 median (Q_1_–Q_3_)	Year 6 median (Q_1_–Q_3_)	*P*
(n/%)	Female	64 (%64.6)	32 (%62.7)	52 (%59.8)	34 (%61.8)	
	Male	35 (%35.4)	19 (%37.3)	35 (%40.2)	21(%38.2)	
Perspective taking		59 (54–63)	58 (52–61)	54 (50–60)	52 (46–55)	*P* < .001†
						p_6-3_ < 0.001†
						p_6-1_ < 0.001†
						p_5-3_ = 0.047*
						p_5-1_ = 0.001*
Compassionate care		43 (39–46)	42 (38–47)	42 (38–44)	39 (34–42)	*P* < .001†
						p_6-5_ = 0.010*
						p_6-3_ < 0.001†
						p_6-1_ < 0.001†
Standing in the patient’s shoes		8 (6–10)	8 (4–10)	8 (7–10)	8 (6–11)	0.100
Total empathy score		108 (100–117)	108 (103–113)	103 (96–109)	98 (87–106)	*P* < .001†
						p_6-5_ = 0.012*
						p_6-3_ < 0.001†
						p_6-1_ < 0.001†
						p_5-1_ = 0.017*
Self-confident approach		20 (18–23)	19 (17–22)	20 (17–21)	20 (17–21)	0.271
Helpless approach		18 (15–21)	21 (18–25)	18 (14–20)	16 (14–19)	*P* < .001†
						p_6-3_ < 0.001†
						p_5-3_ = 0.002*
						p_1-3_ = 0.038*
Submissive approach		11 (10–13)	11 (10–13)	11 (10–13)	11 (10–13)	0.841
Optimistic approach		13 (11–15)	13 (10–15)	13 (12–15)	13 (12–14)	0.437
Seeking social support		12 (10–13)	12 (10–13)	12 (10–13)	12 (10–13)	0.775
Depression score		6 (3–9)	6 (4–8)	6 (3–8)	6 (3–8)	0.680
Anxiety score		9 (6–12)	9 (7–13)	7 (5–10)	7 (5–9)	0.002*
						p_6-1_ = 0.036*
						p_6-3_ = 0.05*
						p_5-1_ = 0.04*

HADS = hospital anxiety depression scale, JSPE-S version = the student version of the Jefferson Physician Empathy Scale.

* Correlation is significant at the 0.05 level (2-tailed).

† Correlation is significant at the 0.001 level (2-tailed).

When the Stress Coping Style Inventory scores were evaluated, it was determined that the difference between the years was only in the helpless approach. It was observed that the helpless approach levels of the third year were higher and this difference was statistically significant when compared with the other classes (P_6-3_ < .001, P_5-3_ = .002, P_1-3_ = .038).

When another variable, the depression, and anxiety scores were examined, no significant difference was observed between the years in terms of depression scores, while the anxiety scores showed significant variability between the sixth year and the first and third years (P_6-1_ = .036, P_6-3_ = .05) and between the fifth year and the first year (P_5-1_ = .04) (Table 1).

When the correlation level between the scales was evaluated, there was a linear and significant correlation between the self-confident approach score and perspective taking (*R* = 0.182, *P* = .002), standing in the patient’s shoes (*R* = 0.172, *P* = .003), and total empathy score (*R* = 0.169, *P* = .004). An inverse correlation between the helpless approach score and the standing in the patient’s shoes score takes attention (r = −0.140, *P* = .017). While the anxiety level of the students did not show any significant correlation on the empathy scale, the depression score showed an inverse correlation with the standing in the patient’s shoes score (r = −0.121, *P* = .039) (Table 2).

**Table 2:** Correlations of JSPE-S version, styles of coping inventory and HADS scores.

Spearman correlation		Self-confident approach	Helpless approach	Submissive approach	Optimistic approach	Seeking social support	Depression score	Anxiety score
Perspective taking	r	**,181** †	,106	,034	,112	−,043	,002	,040
	p	**,002**	,070	,562	,056	,464	,968	,493
Compassionate care	r	,064	,055	,011	,058	−,080	−,016	,028
	p	,272	,353	,853	,321	,173	,781	,636
Standing in the patient’s shoes	r	**,172** †	−,140*	−,075	,085	,113	−,121*	−,089
	p	**,003**	**,017**	,203	,149	,054	**,039**	,131
Total empathy score	r	**,169** †	,049	−,002	,111	−,053	−,038	,005
	p	**,004**	,407	,978	,059	,370	,520	,929
Self-confident approach	r	1000	−,362†	−,205†	,622†	,117*	−**,422**†	−**,370**†
	p	.	,000	,000	,000	,045	**,000**	**,000**
Helpless approach	r	−,362†	1000	,405†	−,351†	−,013	**,371** †	**,606** †
	p	,000	.	,000	,000	,831	**,000**	**,000**
Submissive approach	r	−,205†	,405†	1000	,026	,003	**,191** †	**,266** †
	p	,000	,000	.	,659	,954	**,001**	**,000**
Optimistic approach	r	,622†	−,351†	,026	1000	,090	−**,347**†	−**,441**†
	p	,000	,000	,659	.	,126	**,000**	**,000**
Seeking social support	r	**,117** *	−,013	,003	,090	1000	−**,212**†	−,076
	p	**,045**	,831	,954	,126	.	**,000**	,195

HADS = hospital anxiety depression scale, JSPE-S version = the student version of the Jefferson Physician Empathy Scale.

* Correlation is significant at the 0.05 level (2-tailed).

† Correlation is significant at the 0.01 level (2-tailed).

## 4. Discussion

This research sought to identify the empathy level and its relationship with stress coping styles on medical students. When the data were evaluated, it was found that there was a tendency for empathy levels to decline starting in the fifth year, and that the decline got more pronounced in the sixth year. There was no difference between the years in the ability of standing in the patient’s shoes among the empathy components, and the decrease in empathy in the sixth year was mostly in the perspective-taking component. When the relationship between stress coping styles and empathy was examined, it was determined that the self-confident approach was directly correlated with perspective taking and standing in the patient’s shoes, and there was an inverse correlation between the helpless approach and standing in the patient’s shoes. Although it was determined that the anxiety level decreased towards the upper years, it was observed that depression and anxiety levels did not affect the total empathy level, but affected the style of coping with stress.

In recent years, it has been a frequently observed reality that the skill of empathy, which is one of the skills aimed to be acquired during medical school education, progressively decreases towards graduation.^[8,20–22]^ However, it has been suggested that it is not true to consider empathy alone, and revealing the underlying factors will contribute to medical education.^[23]^ It is known that due to the nature of medical education, it is more stressful than other undergraduate education. As a consequence of this, in the study conducted by Park et al, it was found that the increase in the stress level causes a decrease in the level of empathy. Park et al underscored that directing students to seek social support will cause an increase in the level of empathy.^[24]^ In the study by Sun et al, it was suggested that all of the positive stress coping styles showed a direct correlation with empathy.^[25]^ However, the results of the current study have shown us that seeking social support, which is one of the positive coping styles, is not related to the level of empathy. This result suggests that seeking social support is not sufficient to increase empathy. In cases where clinical empathy is not in question, with social support, it can be expected that the individual’s communication skills will increase and he/she can understand the feelings of the other person more easily. It seems that the physician’s being social is not enough for him/her to understand the patients’ emotions that come with the disease. In other words, while individuals with high empathy skills in social life are more likely to help others and receive stronger social support in return,^[26]^ it is not expected that a physician who is more empathetic to his patient will be more likely to receive social support in return.

A salient outcome of this study is increasing in the self-confident approach, which is one of the positive coping methods, increases both perspective-taking and standing in the patient’s shoes. Therefore, it was seen that the self-confident approach, one of the positive stress coping styles, was more effective on empathy than other positive coping styles. This result confirms the study showing that the increase in the sense of self-sufficiency increases the ability to empathize.^[27]^ The clinician who has problems with self-confidence will have many concerns about the patient approach. At the same time, physician will probably overlook the understanding of the patient’s feelings. However, if it is considered that high self-confidence and low empathy are together in narcissistic personality disorders, it will be concluded that there should be a limit to self-confidence. In last classes, students establish more contact with the patient upon admission to the clinic. For this reason, it is thought that they are worried about taking responsibility and using theoretical knowledge in practice after graduation, and as a result, they experience loss of self-confidence.^[28,29]^ At the end of the this study there was no statistically significant difference between the years in the self-confident approach, it was observed that there was a difference in the interval value. This result may be due to the small sample size.

One of the important findings of the study is that the self-confident approach is correlated with the cognitive (perspective taking) and emotional (standing in the patient’s shoes) components of empathy. Current outcome reveals that the self-confident approach has a more significant effect on empathy. The findings of the study show that perspective-taking, temporarily increases in the first years, then plateaus and declines after the internship, as suggested in the literature.^[30]^ Perspective-taking occurs when a person can think and feel as though they are someone else, rather than themselves. Based on its ability to lessen bias and stereotyping, foster empathy, and enhance interpersonal interactions, perspective taking has the potential for improving healthcare delivery.^[31]^ In order to further develop empathy, the medical education curriculum should incorporate strategies that boost students’ self-confidence. As self-confidence levels drop in the third grade, policies to reduce “clinical competence anxiety” should be promoted.

It has been observed that the helpless approach, a style of coping with negative stress, and the depression scores affect the ability to standing in the patient’s shoes, and there is an inverse correlation between them. In other words, the helpless approach and depression are effective in the emotional component of empathy rather than the cognitive and behavioral component. It is expected that individuals who adopt the helpless approach (because they will feel emotionally weak) have limited emotional empathy skills. Among the years, the helpless approach is most commonly seen in the 3rd-year students. Relatedly, the lowest scores for standing in the patient’s shoes were observed in the 3rd- year students, but this did not cause a statistical difference between the years. In addition, contrary to the hypothesis established at the beginning of the study, it was observed that as the education year increased, there was no increase in the adopted maladaptive defense mechanisms, and even the level of helpless approach decreased even more after the third year. However, the reason why the expected increase in the ability to standing in the patient’s shoes was not observed may be due to the small sample size.

The findings obtained in this study suggest that empathy skills decreased with medical education, especially in perspective-taking and compassionate care components. It was determined that the compassionate care skill was not related to the styles of coping with stress, but the cognitive and emotional components of empathy were affected by the self-confident approach. Focusing on the approaches that improve the students’ self-confidence will have implications for honing empathy. With this regard, it is recommended to boost the approaches such as increasing the contact of the students with the patients and giving an active role in the diagnosis and treatment processes. This point of view indicates that confident medical students will also become empathetic physicians in the future.

## Acknowledgments

The author would like to thank the Afyonkarahisar Health Sciences University students who assisted with the data collection.

## Author contributions

**Conceptualization:** Betül Kurtses Gürsoy.

**Data curation:** Betül Kurtses Gürsoy.

**Formal analysis:** Betül Kurtses Gürsoy.

**Investigation:** Betül Kurtses Gürsoy.

**Methodology:** Betül Kurtses Gürsoy.

**Writing – original draft:** Betül Kurtses Gürsoy.
